# Editors’ Choice 2024

**DOI:** 10.1038/s44172-024-00335-9

**Published:** 2025-01-24

**Authors:** Miranda Vinay, Maria Sakovsky, Alessandro Rizzo, Yasaman Ghasempour, Rosamund Daw, Chaoran Huang, Saleem Denholme, Ali Behnood, Wan-Ting Chen, Or Perlman, Anastasiia Vasylchenkova, Massimo Mastrangeli, Sandra Rugonyi, Jordan Raney

**Affiliations:** 1 Communications Engineering, Springer Nature, Berlin, Germany; 2 Department of Aeronautics and Astronautics, Stanford University, Stanford, CA USA; 3 Department of Electronics and Telecommunications, Politecnico di Torino, Turin, Italy; 4 Department of Electrical and Communications Engineering, Princeton University, New Jersey, USA; 5 Communications Engineering, Springer Nature, London, UK; 6 Department of Electronic Engineering, the Chinese University of Hong Kong, Hong Kong SAR, China; 7 Communications Physics, Springer Nature, London, UK; 8 Department of Civil Engineering, University of Mississippi, Oxford, MS USA; 9 Department of Plastics Engineering, University of Massachusetts Lowell, Lowell, MA USA; 10 Department of Biomedical Engineering and Sagol School of Neuroscience, Tel Aviv University, Tel Aviv, Israel; 11 Department of Microelectronics, TU Delft, Delft, The Netherlands; 12 Department of Biomedical Engineering, Oregon Health & Science University, Portland, OR USA; 13 Department of Mechanical Engineering & Applied Mechanics, University of Pennsylvania, Philadelphia, PA USA

**The editorial team at** ***Communications Engineering*** **highlight a selection of their favourite publications from the journal released in 2024**.

Here we highlight fourteen Research Articles from *Communications Engineering*, telling stories of insight and engineering from across numerous research disciplines that we published in 2024. They are presented in order of publication date from earliest to latest in the year. More Research Highlights are presented in our *Nature Portfolio* collection, which showcases coverage of our content by the *Nature* journals and *Nature*’s multimedia team: https://www.nature.com/collections/commsengcoverage.

**Ultrathin quasi-2D amorphous carbon dielectric prepared from solution precursor for nanoelectronics; Fufei An** **et al.**

2D dielectric materials are vital components of future 3D nanoelectronic devices. Amorphous monolayer carbon is being explored as an alternative for typical bulk metal oxides, for example silicon dioxide (SiO2), and 2D hexagonal boronitride dielectrics. But amorphous carbon is thermodynamically unstable, instead, favoring reconstruction into crystalline phases under typical high temperature 2D material growth conditions. Controlling the thickness precisely while maintaining the amorphous structure is another challenge.

**Fig. 1:**
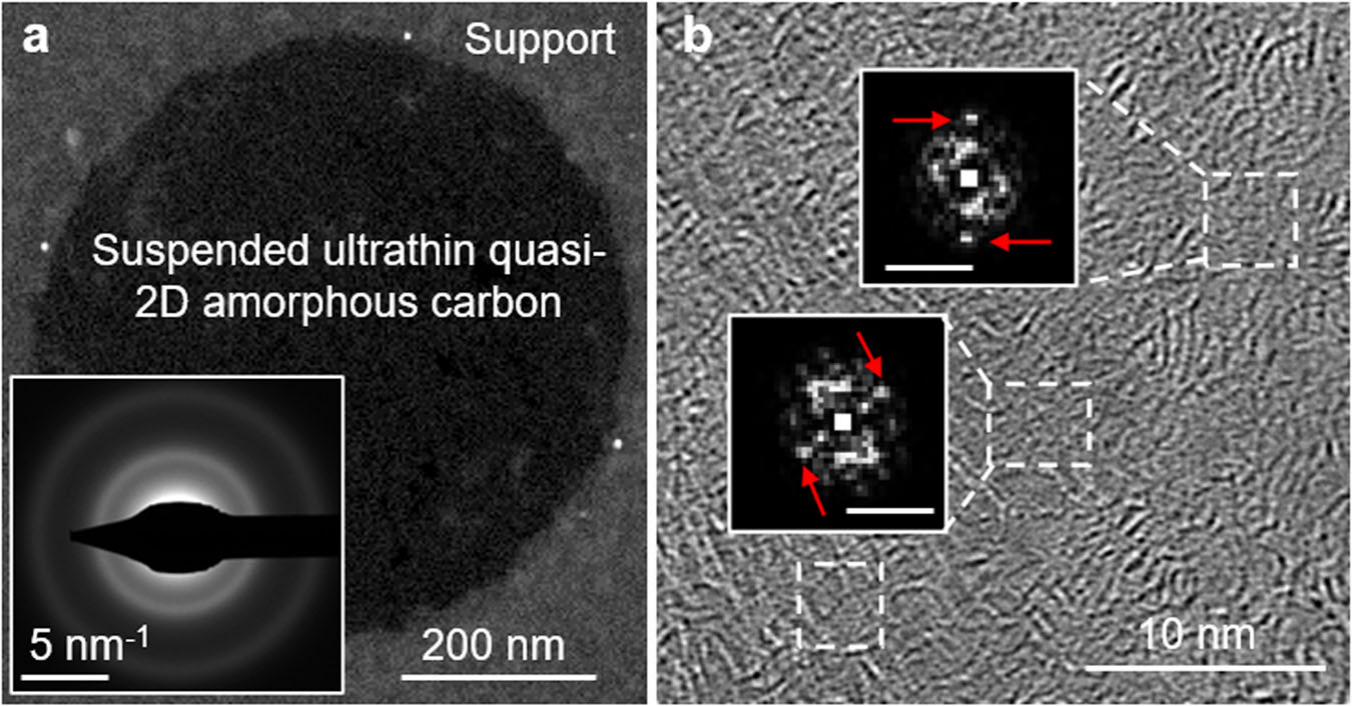
Electron micrographs of freestanding atomically thin quasi-2D amorphous carbon nanomembrane. **a** Low-magnification annular dark-field scanning transmission electron microscope (STEM) image and selected area electron diffraction pattern (inset). **b** Medium-resolution STEM image. Replicated from An et al.^1^.

Current methods for growing amorphous carbon monolayers focus on chemical vapor deposition, which uses additional laser or plasma activation to yield freestanding films at a reduced processing temperature. In a contribution to *Communications Engineering*^1^ (published in December 2023, just too late to make the 2023 Editors’ Choice selection) Funfei An and colleagues report a solution-based approach to fabricate thin layered amorphous carbon films and their multilayered stacks as dielectrics on up to 3-inch wafers. Using dispersed carbon quantum dots as precursors, the researchers describe a scalable, low-temperature solution-based process that creates freestanding membranes consisting of 1 to 2 atomic layers. The carbon dots are mass-produced from high-quality coal char which are assembled into a quasi-monolayer with random orientation under centrifugal force. Annealing at 500 °C under an inert atmosphere then crosslinks the dots together to form a continuous and freestanding film, sealing the inter-dot gaps (Fig. 1). These layers are stable enough to be transferred to different substrates and can be stacked in a layer-by-layer fashion to create precisely controlled multilayer structures, retaining their dielectric properties. As a metric, the resistivity of the dielectric is higher than 1 × 10^9^ Ω⋅cm and the breakdown field is ~ 20 MV⋅cm^−1^. The researchers apply these films as dielectric media in both graphene and molybdenum disulfide (MoS_2_) transistors and as the switching medium in memristor devices.

The amorphous films show interesting performance in these devices. First, the graphene and MoS_2_ transistors show low leakage current and high mobility, compared to transistors made with SiO_2_. Further, they find that the switching voltages and variability of the memristors is reduced. The two dimensional nature of the dielectric also limits the number of atoms involved in modulating the filament formation, thereby increasing the device operating speed and reducing the energy consumption.

The properties of the materials and the device prototypes are interesting. Future work could include deeper materials characterization and further exploration of technological potential. But as it stands, the work offers a versatile solution-deposited approach to create dielectrics for nanoelectronics, with hopes towards even broader applications such as catalysis support and water purification. *Miranda Vinay*

**Soft electronic skin for self-deployable tape-spring hinges; Yao Yao and Xin Ning**

Deployable space structures are critical for space exploration, enabling the large areas required for the operation of solar arrays, communication and radar antennas, and space telescopes. These are packaged into rocket fairings for launch and unfolded once on orbit. Deployables frequently leverage thin-walled structures that undergo large bending deformations for packaging – finely balancing requirements for large functional area, low weight, and high structural rigidity when deployed. However, in-situ sensing of the deformation and vibrations that such structures undergo during deployment and operation remains a challenge.

Yao Yao and Xin Ning report in *Communications Engineering*^2^ a soft electronic skin that monitors deformation, strain, and dynamics in a deployable thin-walled hinge. The skin contains a MEMS-based accelerometer, MEMS-based gyroscope, and three custom-built thin-film strain gauges. The strain gauges are composed of reduced graphene oxide and single-walled carbon nanotubes with a gauge factor of around 50 for improved sensing resolution. This lightweight 0.36 g skin is integrated with the deployable hinge and can undergo large deformations without impacting the hinge’s folding and deployment response. Testing shows the capability of the in-situ sensing method to monitor quasi-static hinge folding and twisting and to characterize dynamic deployment and the structure’s natural frequencies.

This study presents a novel combination of soft electronics with deployable space structures. The results reveal the potential of soft electronics to enable structural health monitoring in space structures without negatively impacting the weight or foldability of the structure. As Yao and Ning suggest, future applications can include in-situ shape reconstruction of large structures on orbit. The next generation of space telescopes and radio-frequency apertures continuously push for larger sizes, leading to highly flexible structures. Efficient shape sensing is therefore an enabling technology for these applications. *Maria Sakovsky*

**Automatic design of stigmergy-based behaviors for robot swarms; Muhammad Salman** **et al.**

Stigmergy, a concept inspired by the way ants communicate through pheromones, is emerging as a powerful tool for programming robot swarms to work together efficiently. However, for many researchers and engineers, designing these collective behaviors manually can be a daunting, time-consuming, and expertise-heavy task. This complexity has been a significant barrier to the widespread adoption of stigmergy-based systems in robotics.

In their paper published in *Communications Engineering* this year, Muhammad Salman and colleagues have introduced an innovative approach to this problem^3^. They developed an automatic design process for stigmergy-based behaviors using Habanero—a general framework embracing optimization algorithms, simulations, and a modular software architecture. This method allows robots to lay and sense artificial pheromones, mimicking natural stigmergy. By simulating four typical coordination scenarios (*aggregation*, *decision making*, *rendezvous*, and *stop*), the system identifies the most effective strategies, which are then tested on real robots. This approach not only reduces the time and expertise required but also enhances the sophistication and adaptability of the robot swarms.

The study demonstrates that automatically designed behaviors can achieve high levels of efficiency and coordination, potentially surpassing those created manually by researchers. This breakthrough could introduce more capabilities in the deployment of robot swarms in applications from environmental monitoring to search and rescue missions. By making the development process more accessible and scalable, this research could expedite a broader use of autonomous robots, accelerating innovation in the field. *Alessandro Rizzo*

**Curving THz wireless data links around obstacles; Hichem Guerboukha** **et al.**

**Fig. 2:**
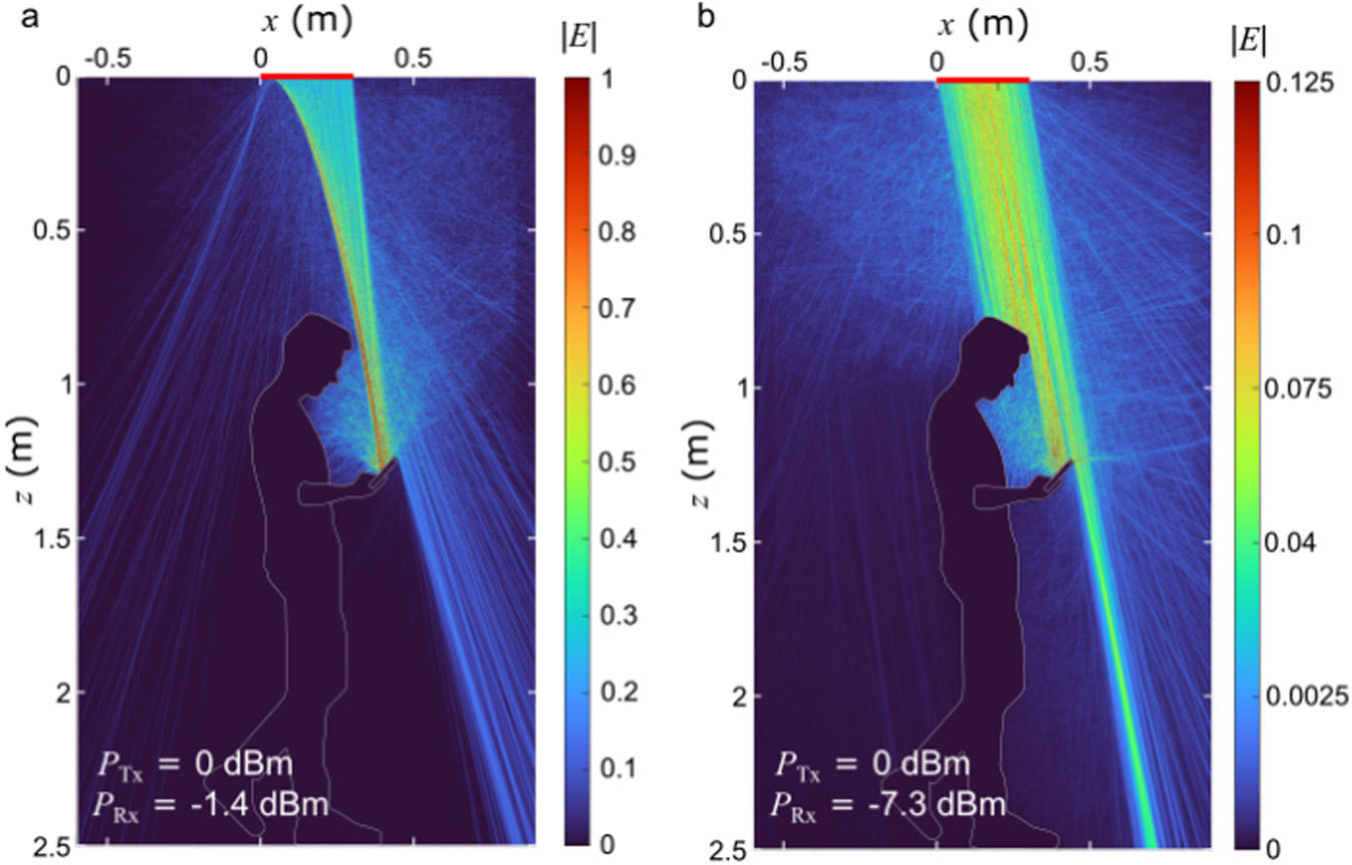
Numerical simulations comparing the transmission of electromagnetic energy from a ceiling-mounted base station to a hand-held cell phone, for two different types of beams. **a** a curved beam, and (**b**) a conventional beam following a straight-line path. The curved beam delivers ~4 times more power to the receiver. Replicated from Guerboukha et al.^4^.

The use of frequencies in the millimeter wave to terahertz band (0.1–1.0 THz) for ultra-fast communication has gained a lot of attention in recent years. Yet despite recent advances across all layers of the communication stack, one challenge has remained unaddressed: the blockage of the line-of-sight path between base stations and users due to the mobility of individuals and objects. This obstruction leads to disruptions in communication and degraded service quality. An article by Hichem Guerboukha and colleagues^4^ published in *Communications Engineering*, suggests a new solution that utilizes the electromagnetic waves in the near field, so not far from the user, to engineer wave fronts capable of maintaining connections despite obstacles. The core innovation involves using self-accelerating beams, which can curve around obstacles while still transmitting data at high bit rates. The ability of such curved beams to increase the amount of delivered power to the user under obstruction is demonstrated in Fig. 2.

This study includes a model to analyze the bandwidth limitations of these beams and experimentally evaluates their ability to carry data-modulated signals. Notably, these beams utilize the entire aperture of the transmitter, including areas that lack a direct line of sight to the receiver, illustrating that traditional ray optics do not adequately describe the behavior of near-field wave fronts.

The researchers emphasize that at terahertz frequencies, the near field range can extend to tens of meters for reasonably sized apertures, making this approach particularly effective for indoor wireless local area networks. Overall, this research not only provides a potential solution for the prevalent issue of blockage in high-frequency wireless networks but also demonstrates the practical advantage for wave front management, aligning with the evolving demands of future communication systems, particularly those targeted for 6 G networks and beyond. *Yasaman Ghasempour*

**Meso-scale seabed quantification with geoacoustic inversion; Tim Sonnemann** **et al.**

In a contribution in *Communications Engineering*, Tim Sonnemann and colleagues report a new method to extract detailed information of the structure of the sea bed from acoustic reflection coefficient data^5^.

Understanding the structure of the seabed can be of importance in a variety of applications. Infrastructure such as offshore wind and tidal turbines, and trans-oceanic communication links require a stable sea floor for reliable anchoring. Seabed knowledge can also be useful to optimise sonar applications for tracking and search activities and understanding marine geohazards.

The acoustic data used to test the technique was originally collected along a 12 km track on the Malta Plateau in the Mediterranean Sea. The set up used an autonomous underwater vehicle towing an acoustic wave source and a linear array of hydrophones to collect incoming acoustic waves. The hydrophones recorded waves rebounding from the surface and sub-surface of the sea bed.

The researchers demonstrated that geoacoustic information of high lateral and vertical resolution was contained in the Bragg interference structure, i.e. the interference pattern between the upward and downward propagating acoustic waves within sediment layers, captured in the acoustic reflection coefficient data.

The sedimentary structure information was extracted by an inversion technique using a sediment acoustic model with quantifiable parameters including porosity, grain-to-grain compressional modulus, and a material index defining strain hardening. These parameters can be used to identify sediment acoustic properties of bulk density, sound speed, and sound attenuation which allow the authors to infer sedimentary structure from their data.

The researchers achieved a resolution of 10 cm in the vertical and 50 m in the horizontal at a depth to 6.8 m below the seafloor, with the method also providing a quantitative estimate of uncertainty. They determined layered features of greater and lesser porosity, which proved to be compacted mud, and mud mixed with sand and shells. They compared their findings to coring measurements, independent sub-bottom profiling, and data fit evaluations to show confidence in their sediment parameter estimates.

This approach achieves subsea acoustic sensing capabilities at a new level of resolution, providing insights into seabed variability for use in diverse marine applications. *Rosamund Daw*

**Can holographic optical storage displace Hard Disk Drives? Jiaqi Chu** **et al.**

The rapid growth of cloud computing and its many applications, such as artificial intelligence, machine learning, and data analytics, are placing enormous pressure on data storage systems. These systems now need to deliver faster access speeds, lower latency, and greater cost efficiency to keep up with demand.

Today, Hard Disk Drives (HDDs) are the leading storage technology because of their low cost. However, HDDs are reaching their physical limits in terms of storage capacity. Their performance is also restricted by the mechanical nature of spinning disks, which has not seen significant improvements in years.

Holographic data storage is a technique in which data is written into a data storage medium as a three-dimensional hologram created by modulating the refractive index of the material; the data can be read back using light beams. Holographic data storage is emerging as a promising alternative that could potentially replace HDDs in cloud storage systems. It offers the benefits of higher storage density and faster access rates, addressing many of the limitations of HDDs. However, a major challenge for holographic storage is ensuring the durability of the stored data over time.

To tackle this issue, Jiaqi Chu and colleagues from Microsoft proposed a media- and workload-aware energy optimization framework that enhances the durability, energy efficiency, and storage density of the rewritable photorefractive medium iron lithium niobate, for warm holographic data storage (data that is not accessed frequently but is still important enough to be kept online, rather than archived offline)^6^. This new optimization framework has achieved a record number of successful reads and set new benchmarks for holographic storage density. These advancements highlight the potential of holographic data storage as a new technology for the future of cloud infrastructure, but also show that more work needs to be done to find holographic media with lower write energy requirements to make this a competitive cloud storage technology. *Chaoran Huang*

**Crash-perching on vertical poles with a hugging-wing robot; Mohammad Askari** **et al.**

Unmanned Aerial Vehicles (UAVs) are increasingly employed for a range of tasks from delivery to inspection, where precise positioning is crucial for their effective operation. Although the aerodynamic efficiency of winged UAVs makes them well-suited for long-distance missions, reliably landing on curved or uneven vertical surfaces remains a significant challenge, particularly in infrastructure inspection. Current solutions often rely on adhesive-based or specialized landing mechanisms, which can be highly sensitive to surface conditions or add substantial structural weight. A study published in *Communications Engineering*^7^ introduced an innovative approach that transforms UAV wings into dual-purpose components, serving not only as flight surfaces but also as robust mechanical systems for securely attaching UAVs to vertical structures, reducing the associated drawbacks and expanding their operational scope.

**Fig. 3:**
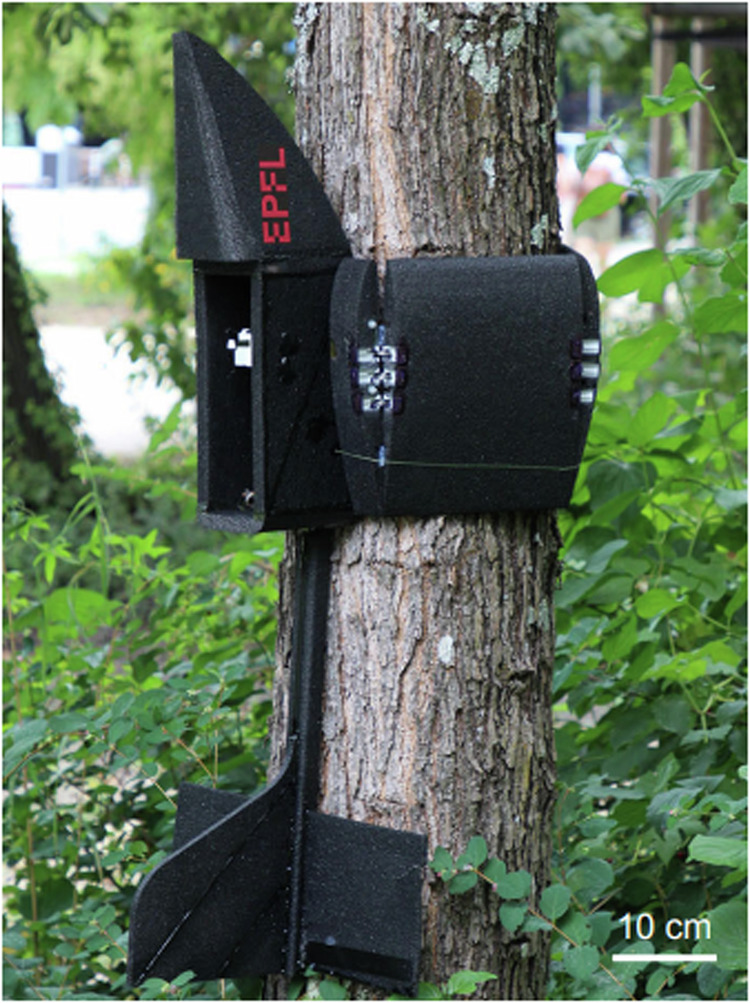
PercHug robot perching vertically on a tree by hugging. Replicated from Askari et al.^7^.

Taking inspiration from the gecko’s crash-landing strategy and the propensity of some winged creatures, such as bats and fledging owls, which wrap their wings around tree trunks to perch vertically, Mohammad Askari and colleagues designed a hugging-wing UAV that upon impact with vertical poles can perch on them (Fig. 3). The same way in which a gecko will land headfirst onto a tree trunk, the UAV is designed with an upturned nose that allows a reorientation of the main body as it comes into contact with the pole at an appropriate angle and high velocity. This suitably positions the robot for its folding wings to then snap around the vertical surface using a latch-release mechanism and hold the UAV in place before it can fall to the ground.

This simple yet effective design is a neat example of bioinspired engineering, adeptly imitating techniques that nature has perfected over eons of evolution. The researchers envisage potential applications in the inspection of complex industrial environments, where vertiginous, small-scale structures are difficult to reach by maintenance personnel. There is also the possibility of in-situ data collection of hard-to-reach wildlife habitats, where human presence would be intrusive. However, advances in targeting capability and the ability to detach from the surface and continue to another destination are crucial next steps for developing such sophisticated applications. *Saleem Denholme*

**Structural integrity of aging steel bridges by 3D laser scanning and convolutional neural networks; Georgios Tzortzinis** **et al.**

Aging steel bridges are critical components of transportation infrastructure, yet they face significant risks from corrosion, which compromises their structural integrity over time. Corrosion not only deteriorates the strength of these structures but also poses severe safety risks, including catastrophic failures that may lead to fatalities, injuries, and disruptions. Currently, inspection practices rely heavily on subjective visual assessments and low-precision tools, resulting in labor-intensive processes that often lack consistency and accuracy^8^. These traditional methods are further constrained by accessibility issues, time-intensive data collection, and an inability to provide detailed evaluations of corrosion patterns and their impacts on structural capacity. The growing demand for safer and more efficient maintenance strategies has highlighted the need for innovative solutions to enhance the accuracy, efficiency, and predictive capabilities of bridge assessments.

**Fig. 4:**
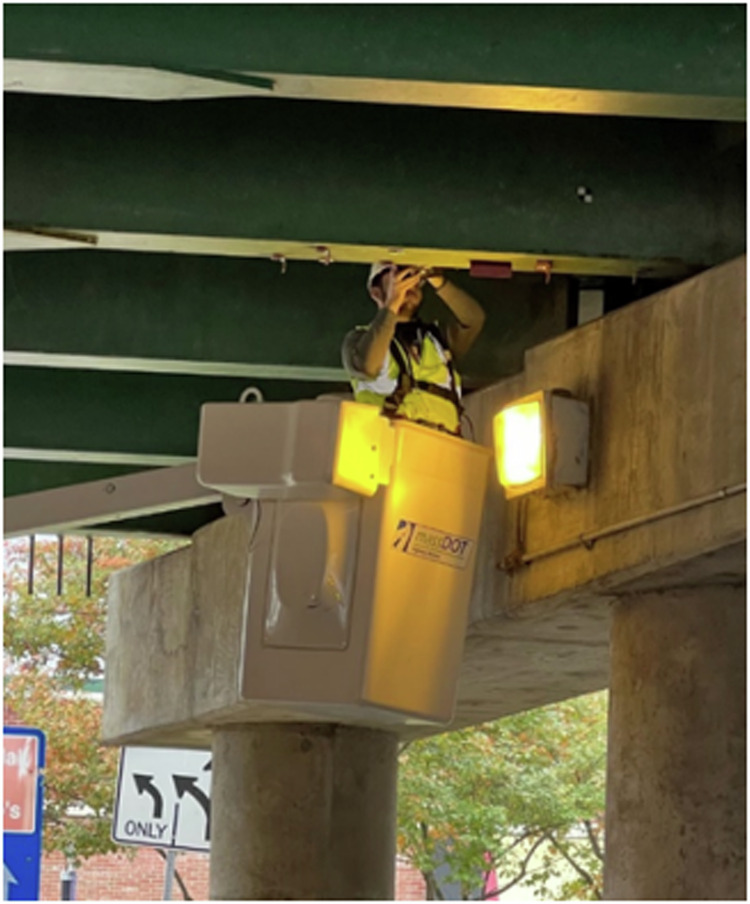
Inspection and structural integrity evaluation of an in-service bridge. Field-based 3D scanning with portable equipment. Replicated from Tzortzinis et al.^9^.

To address these challenges, Georgios Tzortzinis and colleagues presented in *Communications Engineering*^9^, an innovative framework that combines 3D laser scanning and convolutional neural networks (CNNs) for precise evaluation of corroded steel bridges. The study integrates advanced point cloud data from 3D laser scanning with CNNs trained on over 1400 corrosion scenarios, achieving remarkable classification and regression accuracy with errors as low as 2.0% and 3.3%, respectively. The method enables high-resolution visualization of corrosion profiles and accurate predictions of residual structural capacities. Validated on eight decommissioned girders and applied to an in-service bridge, the framework demonstrates transformative potential in bridge maintenance (Fig. 4).

Significantly, the approach replaces labor-intensive, subjective evaluations with a robust, automated system. The findings underscore the viability of modern technologies in revolutionizing infrastructure inspections, paving the way for safer and more cost-effective bridge management strategies. *Ali Behnood*

**Hybrid graphenic and iron oxide photocatalysts for the decomposition of synthetic chemicals; Raphaell Moreira** **et al.**

There are significant environmental and health risks posed by per- and polyfluoroalkyl substances (PFAS). PFAS are synthetic chemicals that resist degradation and persist in the environment and organisms for extended periods. In their *Communications Engineering* paper^10^, Raphaell Moreira and colleagues describe a simple, cost-effective method to synthesize an iron oxide/graphenic carbon (Fe/g-C) hybrid photocatalyst for PFAS degradation. This photocatalyst efficiently degrades perfluorooctanoic acid (PFOA), a common type of PFAS. The Fe/g-C hybrid photocatalyst achieved over 85% degradation efficiency of PFOA at a fluence rate of 1.42 mW/cm^2^ within 3 h in a batch photoreactor system with an initial PFOA concentration of 1 ppm.

This approach improves on prior work by utilizing readily available materials and a straightforward fabrication procedure, enhancing its accessibility and cost-effectiveness for potential commercialization. The Fe/g-C hybrid photocatalyst also demonstrated high degradation rates over extended periods, indicating its stability and suitability for long-term use. Mechanistic insights from the study reveal that the hybrid structure enhances the generation of reactive species under UV light, which efficiently breaks down the strong carbon-fluorine bonds in PFAS. However, one limitation of this approach is that the total fluoride recovery using the 32 wt% Fe/g-C hybrid decreased to 42.2% within 1.5 h and remained relatively constant afterward, possibly due to the adsorption of generated fluoride onto the catalyst surface.

This work offers a promising solution for addressing PFAS contamination in water, contributing to a cleaner and healthier environment. The use of a frugal approach with an abundant heterogeneous hybrid photocatalyst that rapidly decomposes PFOA underscores its potential for impactful environmental remediation. *Wan-Ting Chen*

**Interactive computer-aided diagnosis on medical image using large language models; Sheng Wang** **et al.**

In a year when Nobel Prizes in Physics and Chemistry are tightly linked with artificial intelligence (AI) discoveries, it is hard to underestimate its impact and promise for medicine and engineering. Medical imaging is a common playground for AI deployment, as motivated by the heavy workload set on the radiologist’s shoulder and the historical (yet only 12-year-old) breakthrough in deep learning performance, which arguably started with an image classification challenge^11^.

Large language models (LLMs) are being extensively studied for various biomedical tasks, such as drug discovery, clinical decision support, genomic data interpretation, clinical trial matching, and health informatics. In the radiological context, they are increasingly investigated for generating a textual report from a medical image set.

This year, Sheng Wang and colleagues reported in *Communications Engineering* the development of “chat-computer-aided diagnosis (ChatCAD)”, an interactive system for medical image-based diagnosis using large language models^12^. The system’s backbone intertwined a machine-learning-based report generator with a deep lesion segmentation pipeline and a disease classifier. By appropriate prompt design and training on more than 200,000 chest X-ray images from over 65,000 patients, ChatCAD was able to obtain improved diagnosis accuracy in classifying common thoracic conditions. In addition, the fused information modalities have also improved the appropriateness of the textual report, yet not its conciseness, compared to expert human radiologists. Interestingly, in a Turing-like test, a clinical expert without substantial LLM experience could not tell the difference between ChatCAD output and that of a human expert.

Not surprisingly, the system’s performance was tightly linked to the size of the LLM, where the best-performing model had 175 billion parameters. ChatCAD also offers interactive interaction, which is particularly valuable for alleviating the subject’s stress or uncertainty while obtaining their diagnosis, especially in online healthcare settings.

While the accuracy and reliability of ChatCAD still need to be verified by large-scale future collaborations with clinical experts, the encouraging performance compared to state of the art, together with the compelling, cost-effective interaction between a patient and a medical “agent,” render this conceptual LLM-CAD combination as a strategy worth following. *Or Perlman*

**Multi-channel portable odor delivery device for self-administered and rapid smell testing; Richard Hopper** **et al.**

During the COVID-19 pandemic, many people experienced a temporary loss of smell, and learned from this experience the critical importance of having a sense of smell in everyday life. Importantly, olfactory dysfunction is an indicator of a range of illnesses and has a severe effect on the quality of life. However, automated tools for evaluating and monitoring the ability to smell are not widely available. Existing smell tests typically use multiple felt-tip pens filled with serial odorant dilutions which are manually presented to the patient by a highly specialized nurse. Because of this, the most common smell test – the odour threshold test - is normally a lengthy clinical procedure, with only few commercially available solutions.

The most common automated solution for odour delivery is the so-called olfactometer - a system of targeted delivery or odour stimuli from liquid or gas odorant to the patient’s nose. Olfactometers comprise of many specialised subcomponents, such as gas cylinders, mixing chambers, pumps and valves, making the system expensive, bulky and difficult to deploy in clinical settings.

In their *Communications Engineering* paper, Richard Hopper and colleagues proposed a portable device for delivering the odour threshold test which can be used for rapid smell testing in clinics and by patients^13^. Their device allows varying concentrations of odorant components to be administered by the use of plug in replaceable cartridges filled with odorant-soaked sponge absorbers. During the trial with patients, the approach took, on average, less than half the time to implement when compared to a standard “Sniffin’ Sticks” test.

The odour delivery system integrates digital control and recording of the patient’s smell perception through a mobile app, removing the need for clinical staff to assist the patient during the test. Moreover, the app-based approach enables instant storage and analysis of the results, so the patient can observe the progress of their smell symptoms. The analysis of clinical performance is currently ongoing.

With the demonstration of the rapid self-administered smell test, the researchers make a case for the importance of introducing olfactory monitoring in a self-care routine, which would potentially allow for medical intervention to treat smell loss, or enable the detection of the early signs of disease, for example, Alzheimer’s disease. *Anastasiia Vasylchenkova*

**A microphysiological system for studying barrier health of live tissues in real time; Ryan Way** **et al.**

The study of physiology in vitro has seen remarkable progress in recent years. This has happened notably thanks to the development of synthetic three-dimensional microenvironments whereby cell constructs, such as tissues or organoids, are hosted and subjected to chemical conditions and dynamic stimulation representative of the in vivo counterparts. Realistic physiological recapitulation is the primary goal of such microphysiological systems, which goes along with continuous quantification of relevant parameters of both microenvironment and biology. The latter requirement is motivating significant efforts in sensor integration within microphysiological systems. In this respect, the work of Ryan Way and colleagues^14^ stands out as a clever instantiation of a microfluidic three-dimensional device designed to host tissue barriers, subject them to controlled fluidic shear stress and oxygen gradient conditions, and evaluate key properties of the barrier in real-time, such as its tightness and viability, by measurement of its transepithelial-endothelial resistance (TEER). TEER sensing is implemented by means of two pairs of gold electrodes. The supporting, custom-designed electronics injects constant-amplitude AC current across one pair of electrodes – and hence, across the tissue barrier – and reads out the voltage drop across the other electrode pair as function of the input frequency. This way the system determines the electric impedance of the tissue, which relays information about both paracellular and transcellular transport across the barrier. The authors tested the system with ex vivo mouse colon, notably demonstrating tissue viability for over 72 hours, and showing the reliability of the impedance measurements through controlled chemical disruption of the tissue. In addition, the multi-layered device is oriented vertically during operation to facilitate removal of bubbles from the microfluidics; and up to three devices can be operated at once through the custom electronics to expand the throughout of the system. Way *et al ‘s* microphysiological system is a remarkable display of microfluidically- and electronically-enhanced in vitro technology for the further development of pre-clinical tissue-based studies. *Massimo Mastrangeli*

**Computational multiphysics modeling of radioactive aerosol deposition in diverse human respiratory tract geometries; Ignacio R. Bartol** **et al.**

While radiation exposure is rare, it can happen under diverse scenarios that could affect both adult and pediatric populations. Quantification of radioactive exposure in human populations is therefore important to prepare for and adequately respond to radiation exposure events.

The evaluation of radioactive aerosol exposure heavily relies on mathematical modeling that uses idealized models of the human respiratory tract (HRT). These models lack physiological specificity as they aim to represent an ‘average’ individual, without consideration of diverse population characteristics, such as gender, age, and importantly, morphological HRT differences in the population. To address this shortcoming, Ignacio Bartol and colleagues^15^ reconstructed subject-specific HRT anatomies from the CT-scans of 542 individuals who were 2–90 years of age, with almost equal representation of males and females. In the first part of the study, they used these reconstructed anatomies and machine learning (Random Forest Regression) to determine geometrical anatomical features that characterized a diverse population, followed by a clustering technique (k-means) to group the subject-specific anatomies into clusters (8 per gender) with distinct anatomical characteristics (e.g. trachea diameter, bronchial angle). Based on this clustering, they selected one representative 3D HRT anatomy from each cluster for further modeling.

**Fig. 5:**
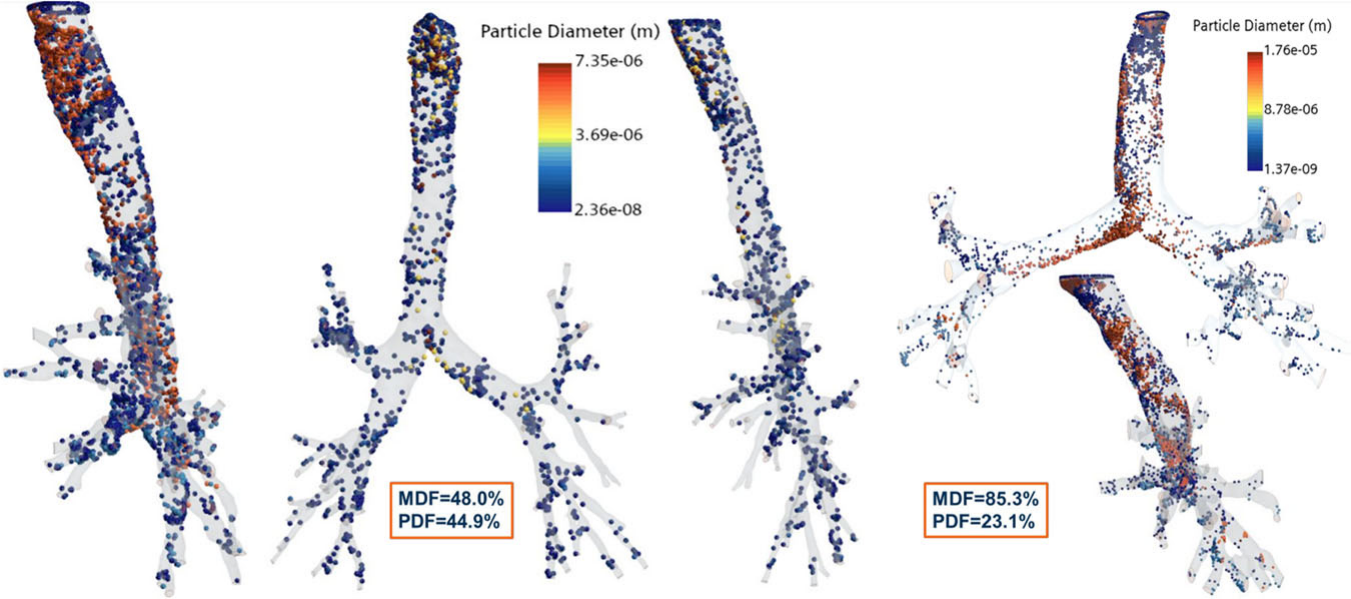
Particle deposition profiles at the end of the second simulated respiratory cycle (physical time = 4 s) in the lower respiratory tract. Mass Deposition Fraction (MDF) and Particle Deposition Fraction (PDF) for three different anatomies are depicted, with striking differences apparent. Replicated from Bartol et al.^15^.

The second part of the study used computational fluid and particle dynamics simulations of the representative 3D anatomies to quantify respiratory airflow and transport of aerosol particles into the HRT. CFPD simulations were used to generate particle deposition profiles among distinct HRT anatomies. A Monte Carlo radiation transport model was then used to quantify absorbed radiation dose within the lungs for the different HRT anatomies. The study found variations in particle deposition profile patterns and radiation dose that strongly depended on HRT anatomy, with even minor discrepancies in HRT anatomy having a large impact on particle deposition patterns (see Fig. 5). The results emphasize the importance of considering diverse HRT population anatomical characteristics when estimating radioactive exposures, and the need for individualized dosimetry models for therapeutic intervention. *Sandra Rugonyi*

**Chain-based lattice printing for efficient robotically-assembled structures; Zhe Xu and Aaron Dollar**

Lattice structures have been long studied for their useful mechanical properties and efficient use of materials. By placing materials just where they will contribute most to key properties such, as stiffness, strength, and toughness, one can build structures that meet performance requirements while simultaneously minimizing weight.

In a publication in *Communications Engineering*, Zhe Xu and Aaron Dollar introduce “Chain-Based Lattice Printing (CLaP)”, an approach leveraging a modular chain of interlinked nodes to create efficient lattice structures^16^. The strategy uses a robot extruder, similar to a 3D printer, but one that extrudes a pre-existing chain of struts. The robot assembles these struts into a user-defined lattice. In principle, CLaP could work with a wide variety of materials, and assemble structures up to meter scales.

Another unique aspect of the authors’ work is that CLaP structures can be disassembled, and components reused for other lattice configurations. This feature could be useful for applications in space exploration, transportation, remote construction, etc. Moreover, since the robot uses pre-existing components, structures can be assembled much more quickly, and with much less energy, than with traditional additive manufacturing, which requires polymerization or phase changes to build structures from material precursors.

This work represents an important step forward in scalable manufacturing of structures, merging the modularity of robotics with the precision of digital fabrication. The potential for lightweight, material-efficient structures opens doors to innovation in a variety of fields. This paper is a must-read for engineers seeking advanced, sustainable solutions in structural design and manufacturing. *Jordan Raney*
